# Pediatric Sleep Quality and Parental Stress in Neuromuscular Disorders: Descriptive Analytical Study

**DOI:** 10.2196/56667

**Published:** 2025-01-28

**Authors:** Sajjad Khaksar, Mehdi Jafari-Oori, Forogh Sarhangi, Malihe Sadat Moayed

**Affiliations:** 1 Student Research Committee, Baqiyatallah University of Medical Sciences, Tehran, Iran; 2 Nursing Care Research Center, Clinical Sciences Institute, Nursing Faculty, Baqiyatallah University of Medical Sciences, Vanak SquareTehran, Iran, 98 9127297199

**Keywords:** spinal muscular atrophy, neuromuscular disorders, sleep quality, pediatrics, parental stress, children, parents, muscular atrophy, muscular disorders

## Abstract

**Background:**

Neuromuscular disorders (NMDs) constitute a heterogeneous group of disorders that affect motor neurons, neuromuscular junctions, and muscle fibers, resulting in symptoms such as muscle weakness, fatigue, and reduced mobility. These conditions significantly affect patients’ quality of life and impose a substantial burden on caregivers. Spinal muscular atrophy (SMA) is a relatively common NMD in children that presents in various types with varying degrees of severity.

**Objective:**

This study aimed to evaluate the sleep quality of children with NMDs, particularly SMA types 1, 2, and 3 and assess the stress levels experienced by their parents.

**Methods:**

A descriptive analytical study was conducted from February to October 2023, in selected hospitals and dystrophy associations in Tehran and Isfahan, Iran. A total of 207 children aged 1‐14 years with various NMDs were included in the study. Data were collected using a web-based questionnaire with 3 parts: demographic information, the Children’s Sleep Habits Questionnaire to assess children’s sleep, and the Stress Response Inventory to measure parental stress. Statistical analyses were performed using SPSS version 22, with an α level of .05.

**Results:**

Significant differences in sleep quality were found among SMA types, with mean scores of 74.76 (SD 7.48) for SMA type 1, 76.4 (SD 7.29) for SMA type 2, 72.88 (SD 6.73) for SMA type 3, and 75.87 (SD 5.74) for other NMDs (*P*=.02). A correlation was found between sleep and length of hospital stay (*r*=0.234, *P*<.001)and between sleep and the child’s sex (*r*=−0.140, *P*=.04). Parental stress scores averaged 95.73 (SD 32.12). There was not a statistically significant difference in parental stress scores among the 4 groups (*P*=.78). This suggests that parental stress levels were similar across different NMD groups.

**Conclusions:**

Sleep disorders are prevalent among children with NMDs, especially SMA. Parents experience high levels of stress that can affect the care they provide. Therefore, interventions to improve children’s sleep and address parental stress are crucial. Regular screening, counseling, and tailored support are recommended to enhance the well-being of children with NMDs and their families.

## Introduction

Neuromuscular disorders (NMDs) are a diverse group of disorders that affect motor neurons, neuromuscular junctions, and muscle fibers, resulting in various disease onsets, presentations, and prognoses. Examples of NMDs include spinal muscular atrophy (SMA), Charcot-Marie-Tooth disease, congenital myasthenia gravis, and Duchenne muscular dystrophy [1,2]. Children with NMDs can also develop central nervous system disorders such as cerebral palsy and spinal cord injury [3]. Common symptoms of NMDs include muscle weakness, fatigue, reduced mobility, and decreased physical performance. Additionally, these patients may experience orthopedic, cardiac, infectious, and respiratory problems, which can negatively impact their quality of life [4].

The global prevalence of neuromuscular diseases, as estimated through a systematic reviews of studies, ranges from 16 per 10,000 to 25.1 per 100,000 individuals and affects people of all ages [5]. The most common autosomal recessive disorder in children with NMDs is SMA, and it affects approximately 1 in 10,000 individuals and has a carrier frequency of 1 in 50 in certain populations [4]. Despite this, there have been no comprehensive epidemiological studies conducted on children with NMDs, particularly SMA, in Iran. Only one study in Iran identified Duchenne muscular dystrophy as the most prevalent NMD, with no comprehensive data available on SMA or other NMDs [6].

Many NMDs, including SMA, cause progressive muscle weakness that affects the respiratory system, leading to reduced upper airway function, impaired coughing and secretion clearance, and weakened chest wall support [7]. As a result, children with NMDs, particularly SMA, are at high risk for upper airway obstruction, pulmonary aspiration, frequent respiratory infections, sleep-disordered breathing (SDB), hypoventilation, and respiratory failure [8,9].

SDB is a prevalent complication in children with advanced NMDs [10]. It occurs intermittently due to partial or complete upper airway obstruction, leading to disrupted sleep patterns and ventilation [11]. The prevalence of SDB in healthy children is approximately 1%, while up to 70% of children with NMDs experience it [12]. The common issues faced by these children include sleep disturbance, drowsiness, night sweats, nausea, morning headaches, fatigue, and poor academic performance. Therefore, effective management of SDB is crucial to reduce complications and enhance the quality of life for children with NMDs [13,14]. According to research, sleep disorders in children can result in sleep problems for their parents. When children struggle to fall asleep, their parents also have difficulty sleeping, which can cause stress and lead to missed workdays [15]. In a recent study, the mental health of parents of a child with a NMD was assessed using the Psychological Adaptation Scale questionnaire, which revealed high levels of mental health problems among parents [16].

Stress and anxiety among parents and caregivers can also have negative effects on children, potentially leading to a lack of support from mothers. Confusion in parental behavior, particularly from mothers, can be harmful to their children [17]. Furthermore, research has indicated that changes in a mother’s psychological functioning, such as increased stress and anxiety, can influence her perception of her child’s sleep problems [18]. Despite numerous studies highlighting the association between parental stress and sleep quality in children with NMDs, comprehensive research specifically focusing on a large cohort of patients with SMA remains limited. Globally, existing studies have primarily concentrated on genetic, laboratory, and epidemiological aspects with small sample sizes and a restricted focus on a few NMDs [6,12,19].

This study aimed to bridge this knowledge gap by examining various aspects of sleep in Iranian children with NMDs, with a particular emphasis on SMA. Using child-specific sleep assessment tools, this study sought to identify the correlation between sleep disturbances and parental stress in this population. The findings of this study can increase our understanding of the sleep experiences of children with NMDs and their parents’ stress levels. Eventually, these findings can be used to formulate approaches that enhance the well-being of such children and minimize emotional strain on parents in various cultural environments.

## Methods

### Study Design and Participants

This descriptive analytical study was conducted as part of a larger study in selected hospitals in Tehran and Isfahan, as well as the dystrophy association of these centers, from February to October 2023, in Iran. The study included a sample of 207 children diagnosed with a NMD, with inclusion criteria of having any muscular dystrophy with an unknown cause and being between preschool and school age (1 to 14 years old). The exclusion criterion was an incomplete questionnaire.

### Data Collection

A cross-sectional web-based survey was conducted using the SurveyHeart platform [20] to collect data from caregivers of children with NMDs. Participants were recruited through convenience sampling at selected hospitals and centers in Tehran and Isfahan, Iran. To optimize participation, caregivers were informed about the aims of the study and the significance of sleep for children with NMDs. Data were gathered using a 3-part web-based questionnaire. The initial section captured the demographic information using closed-ended questions. Subsequently, children’s sleep habits and parental stress levels were assessed using the Children’s Sleep Habits Questionnaire (CSHQ) and the Stress Response Inventory (SRI), respectively, and both used Likert scale items.

### Web-Based Questionnaire

#### Demographic Characteristics

The demographic data examined in this study included the child’s sex and age, number and length of hospitalizations, use of specific medications for treatment, parents’ educational levels, parents’ job, recruitment organization (military or civilian), and number of children in the family.

#### Children’s Sleep Habits Assessment

The CSHQ, which was reviewed and designed by Owens et al [21], was created to assess the sleep habits of 623 preschool- and school-aged children. They showed that the CSHQ was an effective tool for evaluating sleep quality in children. The questionnaire consisted of 35 statements rated on a 3-choice Likert scale across 8 categories: sleep resistance, sleep anxiety, parasomnia, breathing disorders during sleep, waking up at night, sleepiness during the day, sleep duration, and sleep onset delay. Statements 1, 2, 9, 10, and 28 were scored in reverse order. The total score ranged from 33 to 99, with a higher score indicating poorer sleep quality (score ≥41) [21]. In a previous study, the homogeneity of the questionnaire was determined to have a Cronbach α of 0.8 [22]. The validity of the children’s sleep habits questionnaire was assessed based on content validity, and its internal consistency has been found to be 0.82 [15]. In another study, the Cronbach α was 0.816 [23].

#### Parental Stress Assessment

To assess parental stress levels, the SRI scale developed by Koh et al [24] was used. This questionnaire was designed to explore the emotional, physical, cognitive, and behavioral aspects of stress responses. It was a self-reported measure, requiring participants to indicate the extent to which they experience each symptom on a 5-point Likert scale ranging from “not at all” (0 points) to “completely” (4 points). The stress response questionnaire consisted of 39 items and 7 subscales: tension (6 items), aggression (4 items), somatization (3 items), anger (6 items), depression (8 items), fatigue (5 items), and frustration (7 items). The following points were assigned to calculate the score for each tension subscale: 16 for aggression, 12 for somatization, 24 for anger, 32 for depression, 20 for fatigue, and 28 for frustration. The minimum and maximum scores were 0 and 156, respectively. The reliability of the SRI tool was examined, resulting in a Cronbach α of 0.97, with a 3-week interval between assessments [24]. The validity of the Persian version of the SRI tool in Iran was confirmed, with an α coefficient of 0.963. Validity was further assessed through factor analysis using the principal parts method and Varimax rotation [25]. In the present study, the Cronbach α for this tool was 0.941.

### Ethical Considerations

This study was conducted in accordance with the ethical principles outlined in the Declaration of Helsinki. The study protocol was approved by the Ethics Committee of the Baqiyatullah University of Medical Sciences (code IR. BMSU. BAQ. REC.1401.129). Written informed consent was obtained from the guardian or legal guardian of each child participant. Participants (or their legal representatives) had the right to withdraw from the study at any time without any consequences. All data collected during this study was anonymized to ensure participant privacy.

### Statistical Analysis

For statistical analyses, mean tests with SDs and nonparametric tests (Spearman, Kendall *τ*b, and Kruskal-Wallis) were used to measure qualitative and quantitative variables and determine their relationship with the types of dystrophy, respectively. All analyses were considered statistically significant at an α level of .05. The statistical data were analyzed using SPSS version 22 (IBM Corp).

## Results

### Demographic Characteristics

This study aimed to investigate the demographic and clinical characteristics of children with NMDs. Of the 207 children enrolled, 50 (24.2%) had SMA type 1, 95 (45.9%) had SMA type 2, 54 (26.1%) had SMA type 3, and only 8 (3.9%) had other NMDs. Specifically, 4 children had Duchenne muscular dystrophy and 4 children had Becker muscular dystrophy. Regarding sex, 114 participants (55.1%) were boys and 93 (44.9%) were girls. Those with SMA type 1 included 20 boys (9.7%) and 30 girls (14.5%). The children with SMA type 2 group included 50 boys (24.2%) and 45 girls (21.7%), and those with SMA type 3 included 36 boys (17.4%) and 18 girls (8.7%). Additionally, for children with other NMDs, there were 8 boys (3.9%). The mean age of the children was 7.14 (SD 4.41) years. Additionally, out of 207 families, 49 (23.7%) were military, while 158 (76.3%) were civilians (Tables1  2).

Furthermore, we examined the clinical characteristics of these children. Children with SMA type 1 had the longest average length of hospitilization and the highest average number of hospitalizations among the 4 groups. There were significant differences in both the length of stay and number of hospitalizations among the 4 NMD groups (*P*<.001). Conversely, no significant correlations were found between the other demographic variables and the different NMD groups, suggesting that the length of hospital stay varied significantly (Table 2). Additionally, this study assessed the use of specific medications to treat these children. Of the children enrolled in the study, 70% (35/50) with SMA type 1, 63% (60/95) with SMA type 2 (63%), 52% (28/54) with SMA type 3, and 50% (4/8) with other NMDs received disease-specific treatment.

**Table 1.** Demographic characteristics of the study participants (n=207).

Characteristics	SMA^a^ type 1	SMA type 2	SMA type 3	Other NMD^b^	Total
NMD of the child, n (%)	50 (24.2)	95 (45.9)	54 (26.1)	8 (3.9)	207 (100)
Father’s education, n (%)					
Less than a diploma	18 (8.7)	16 (7.7)	19 (9.2)	0 (0)	53 (25.6)
Diploma	16 (7.7)	31 (15)	17 (8.2)	6 (2.9)	70 (33.8)
Bachelor’s degree	12 (5.8)	26 (12.6)	13 (6.3)	2 (1)	53 (25.6)
Graduate	4 (1.9)	18 (8.7)	5 (2.4)	0 (0)	27 (13)
No answer	0 (0)	4 (1.9)	0 (0)	0 (0)	4 (1.9)
Mother’s education, n (%)					
Less than a diploma	12 (5.8)	24 (11.6)	22 (10.6)	0 (0)	58 (28)
Diploma	26 (12.6)	33 (15.9)	24 (11.6)	2 (1)	85 (41.1)
Bachelor’s degree	10 (4.8)	29 (14)	8 (3.9)	4 (1.9)	51 (24.6)
Graduate	2 (1)	9 (4.3)	0 (0)	2 (1)	13 (6.3)
Father’s job, n (%)					
Recruitment	14 (6.8)	30 (14.5)	24 (11.6)	4 (1.9)	72 (34.8)
Part-time	21 (10.1)	38 (18.4)	14 (6.8)	4 (1.9)	77 (37.2)
Home	0 (0)	2 (1)	0 (0)	0 (0)	2 (1)
Unemployed	13 (6.3)	10 (4.8)	15 (7.2)	0 (0)	38 (18.4)
Vacation	2 (1)	15 (7.2)	1 (0.5)	0 (0)	18 (8.7)
Mother’s job, n (%)					
Recruitment	2 (1)	20 (9.7)	5 (2.4)	2 (1)	29 (14)
Part-time	0 (0)	10 (4.8)	2 (1)	0 (0)	12 (5.8)
Home	44 (21.2)	57 (27.5)	43 (20.8)	6 (2.9)	150 (72.5)
Unemployed	2 (1)	0 (0)	0 (0)	0 (0)	2 (1)
Vacation	2 (1)	6 (2.9)	4 (1.9)	0 (0)	12 (5.8)
Recruitment organization, n (%)					
Military	11 (5.3)	23 (11.1)	13 (6.3)	2 (1)	49 (23.7)
Civilian	39 (18.8)	71 (34.3)	41 (19.8)	6 (2.9)	158 (75.8)
Number of children in the family, n (%)					
1	17 (8.2)	44 (21.2)	17 (8.2)	4 (1.9)	82 (39.6)
2	21 (10.1)	34 (16.4)	22 (10.6)	4 (1.9)	81 (39.1)
3	12 (5.8)	15 (7.2)	13 (6.3)	0 (0)	40 (19.3)
4	0 (0)	2 (1)	2 (1)	0 (0)	4 (1.9)

a SMA: spinal muscular atrophy.

b NMD: neuromuscular disorder.

**Table 2.** Demographic characteristics of the study participants.

Variable	SMA^a^ type 1, mean (SD)	SMA type 2, mean (SD)	SMA type 3, mean (SD)	Other NMD^b^, mean (SD)	*P* value^c^
Sleep score^d^	74.76 (7.48)	76.40 (7.29)	72.88 (6.73)	75.87 (5.74)	.03
Parental stress score^e^	94.72 (28.83)	98.81 (30.76)	94.89 (33.69)	91.25 (45.03)	.78
Length of stay (days)	1.76 (0.71)	1.03 (0.19)	1.58 (0.69)	1 (0.00)	<.001
Number of hospitalizations	2.16 (0.87)	1.55 (0.83)	2.09 (0.87)	1 (0.00)	<.001
Age of the child (years)	4.32 (3.65)	9.75 (4.86)	6.25 (4.62)	8.25 (4.62)	.02

a SMA: spinal muscular atrophy.

b NMD: neuromuscular disorder.

c Kruskal-Wallis test.

d Total sleep score: mean 74.37, SD 7.14.

e Total stress score: mean 95.73, SD 32.12.

### Sleep Habits of Children

The mean sleep score for each group was 74.76 (SD 7.48) for SMA type 1, 76.4 (SD 7.29) for SMA type 2, 72.88 (SD 6.73) for SMA type 3, and 75.87 (SD 5.74) for other NMDs. There was a statistically significant difference in the sleep scores among the 4 groups (*P*=.02). This indicated that at least 1 group had a significantly different mean sleep score than the other groups (Table 2). Pediatric sleep was influenced by various demographic variables, some of which had significant correlations while others did not. A significant positive correlation was observed between sleep score and the length of hospital stay (*r*=0.234, *P*<.001), suggesting that longer hospital stays were associated with a decrease in the quality of pediatric sleep. Furthermore, a significant negative correlation was found between sleep and sex (*r*=−0.140, *P*=.04), suggesting that sex differences affected pediatric sleep patterns. However, the correlations between sleep and NMD (*r*=0.121, *P*=.08) and the father’s education (*r*=−0.119, *P*=.08) were weak and nonsignificant. Similarly, the correlation between sleep and the number of children (*r*=.025, *P*=.72) was very weak and nonsignificant, indicating little to no association (Table 3).

**Table 3.** Correlation analysis between pediatric sleep, parental stress, and demographic variables.

Variable		NMD^a^	Length of stay	Parental stress	Father education	Sex	Child number
Pediatric sleep							
*r*^b^		0.121	0.234	0.454	–0.119	–0.140	0.025
*P* value		.08	<.001	.53	.08	.04	.72
Parental stress							
*r*		0.231	–0.049	—	–0.061	–0.017	0.032
*P* value		.46	.48	—	.38	.80	.65

a NMD: neuromuscular disorder.

b Spearman rank correlation coefficient.

### Parental Stress

The parents of children with SMA type 2 reported the highest mean stress score of 98.81 (SD 30.76), followed by the parents of children with SMA type 3 (mean 94.89, SD 33.69), SMA type 1 (mean 94.72, SD 28.83), and other NMDs (mean 91.25, SD 45.03). There was not a statistically significant difference in parental stress scores among the 4 groups (*P*=.78). This suggests that parental stress levels were similar across the different NMD groups (Table 2). No significant correlations were found between parental stress and the demographic variables examined (Table 3).

## Discussion

### Principal Findings

This study found that children with NMDs, especially those with SMA, had significantly lower sleep quality according to the CSHQ. Frequent sleep disturbances in children with NMDs can significantly increase the overall disease burden for patients and their caregivers [26]. As a result, parents of children with NMDs can experience high levels of stress. However, sleep disorders in people with NMDs, especially in children with SMA, have not been well studied. Therefore, it is necessary to evaluate sleep in patients with NMDs [27,28]. Our study is the first to examine a large group of children with NMDs, particularly SMA, in Iran. One notable difference between our study and others [29-33] was the number of patients with SMA. In this study, we assessed the sleep of 199 children with SMA using the CSHQ. The results of our study, demonstrating reduced sleep quality in children with SMA, align with those of Chiang et al [33], who reported significantly lower mean sleep scores in this population compared to healthy controls [34]. These findings are consistent with those of a study on children with SMA type 1 [35], a study of 85 children with SMA type 1 and 2 in Italy [29], a study on 31 children with SMA type 1, 2, and 3 [30], and a study on children with myotonic dystrophy [36].

Furthermore, the results of our study showed that the mean score of sleep disorders in children with SMA type 2 was higher than that of other types of NMDs, although there was not a statistically significant relationship between individual and family factors. However, in contrast to the study by Pera et al [29], sleep disorders were reported more frequently in children with SMA type 2 compared to other children. Additionally, Chacko et al [30] reported fewer sleep disorders in children with SMA type 3. This discrepancy may be attributed to sample size, as the studies done by Chacko et al [30] and Pera et al [29] included only 9 and 13 children with SMA type 3, respectively. Another difference between our study and the aforementioned studies was the use of a sleep assessment tool. Chacko et al [30] used polysomnography, whereas Pera et al [29] used the Sleep Disturbance Scale for Children. However, further research and evaluation are necessary to gain a better understanding of the sleep disorders in children with NMDs.

Sleep problems are common in childhood and adolescence and are related to various factors, such as learning, memory, and emotional and behavioral problems [37-39]. This study aimed to investigate the individual and family factors that influence children’s sleep. The study examined the child’s age, the number and duration of hospitalizations, the parents’ education and occupation, their employing organization, and the number of children in the family. In this study, no significant relationship was found between parents’ education and their children’s sleep. However, a study on children with NMDs found that higher levels of parental education and income were associated with a reduced care burden for parents. This, in turn, led to improved sleep quality and a better overall quality of life for their children [40]. Furthermore, a study conducted on healthy children showed that children whose parents had a university education were more prone to experiencing sleep issues than children whose parents did not graduate from high school [41].

The child’s age was taken into consideration when studying childcare outcomes. This is because as a child with a NMD ages, parents’ caregiving responsibilities become more challenging due to the progression of the disease. For example, a study conducted in Brazil examined 31 caregivers of children with Duchenne and found that older boys were more likely to be better understood by their caregivers in terms of their needs and care [42].

Another result of this study was the difference among various groups of patients with NMDs in terms of sleep examination, age, duration, and number of hospitalizations. The results of Chacko et al [30] also corroborate our findings in a sleep study of children with SMA types 1, 2, and 3. They demonstrated that sleep quality was lower in children with SMA type 1 than those with SMA type 2 and SMA type 3 [30]. The length of stay and number of hospitalizations varied among children with different types of SMA and NMDs in general. The findings of Lin et al [43] also support the results of this study. In terms of hospitalization, Chan et al [44] revealed that patients with SMA type 1 had more than 10 visits per year, patients with SMA type 2 had 8‐23 visits, and patients with SMA type 3 had 12‐28 visits annually. Regarding hospital stays, the average stay length for patients with SMA type 2 was longer than patients with SMA type 3 but shorter than patients with SMA type 1. The results from Chan et al [44] also confirmed the difference in the duration and number of hospitalizations among these children. Additionally, there was a significant relationship between the duration of hospitalization and sleep in children. When children are hospitalized, they tend to sleep less and have lower quality sleep [42]. A study also found that children admitted to general pediatric and intensive care units slept an average of 2 hours less than they did at home before hospitalization, according to their parents’ reports [42]. It is important to note that admitting a child to a hospital is a stressful experience that can increase parental anxiety.

Parents of a child with a chronic disease often experience significant stress that impacts various aspects of their lives [45]. This study aimed to investigate the stress levels of parents of children NMDs by using 7 subscales: tension, aggression, somatization, anger, depression, fatigue, and frustration. The results indicated that parents of children with NMDs experience varying levels of stress, with parents of children with SMA type 2 experiencing higher levels. This aligns with a study that found no significant difference in stress levels among parents of children with NMDs such as Williams syndrome, Down syndrome, and autism spectrum disorder [46]. This study also stated that the similarity in parental stress levels across different NMD groups suggests that the specific type of disorder may not be the main cause of stress [46]. Additionally, a study reported that parents of children with NMDs experienced high levels of stress, with no significant difference between mothers and fathers [47]. Another study examined emotional distress symptoms among mothers of sons with Duchenne and Becker muscular dystrophy, comparing them to a control group of women matched for sex and age. The study found that these mothers reported a lower quality of life and more emotional distress, depression, stress, and clinical anxiety symptoms compared to the women in the control group [48]. Given the consistent reports of higher stress levels among parents of children with NMD, it is crucial to focus on implementing adaptive strategies for families and parents. Screening and intervention measures for families of children with NMDs are essential steps to support these families. NMD associations that provide assistance to these patients should consider implementing measures to screen and support parents of these families. It would also be beneficial to identify centers that offer mental health services and refer families based on their health insurance coverage.

In addition, we expected to observe a significant correlation between children’s sleep and parents’ stress levels. However, this relationship was not statistically significant, despite several studies indicating a connection between children’s sleep and parents’ stress [18,49,50]. Perhaps this difference can be attributed to the numerous and diverse factors that impact the sleep of children with NMDs and the stress experienced by their parents. Additionally, the intermittent nature of medication administration, frequently resulting from drug supply constraints, can further compromise the sleep quality in this patient population.

Another study aimed to assess the level of stress experienced by parents of children who were hospitalized. The study included 352 parents whose children were hospitalized, and it was found that these parents generally experienced mild-to-moderate levels of stress. Interestingly, the study also revealed that parents who reported lower stress levels tended to feel more satisfied. This study identified several factors that predicted higher levels of stress among parents. These factors included having a low level of education, having a child hospitalized for more than 14 days, and having a child who had visited the hospital frequently in the past [51]. These findings can be generalized to other children with neurodevelopmental disorders because parents of such children often experience higher levels of stress, due to the frequent hospitalizations that their children require. As the duration of their child’s hospitalization increases, parents are more likely to report higher levels of stress.

This study, along with others that emphasize sleep disturbances in children with NMDs and the stress experienced by their parents, highlight the urgent need for comprehensive interventions. In this context, studies by Bedi et al [52] and Mellies et al [53] demonstrated that noninvasive ventilation normalizes nocturnal gas exchange and improves diurnal gas exchange, respiratory disturbance index, arousals from sleep, nocturnal heart rate, sleep architecture, and overall quality of life in children with NMDs by reducing symptoms and enhancing daily functioning. However, adherence to noninvasive ventilation, especially in rapid eye movement–related SDB, remains unclear. For instance, nocturnal bilevel positive airway pressure treatment has been shown to help individuals with limb-girdle muscular dystrophies return to their usual daily activities [54]. In addition to noninvasive ventilation, other interventions such as pharmacological treatments, behavioral strategies, and sleep health literacy are also important [55-57]. Implementing these interventions can be challenging; however, they are crucial for improving sleep quality and overall health. It is essential to understand which patients are at a higher risk of developing sleep dysfunction and should be actively monitored. Moreover, more rigorously designed studies are needed to evaluate the long-term benefits and cost-effectiveness of various sleep interventions for children with NMD.

### Limitations

One limitation of this study was its methodology. The findings reported here were correlational, not causal, and they do not imply causality. Data were collected using self-reported web-based questionnaires. The extensive sample size necessitated the use of this method; however, it limited control over response conditions, thereby introducing potential biases and reducing the reliability of the findings. Our current methodology does not thoroughly evaluate various factors, such as environmental, psychological, physiological, and social influences on sleep quality. Future research should employ a multidimensional approach, incorporating objective sleep measurements and detailed clinical evaluations to gain a more comprehensive understanding of the sleep disorders in this population.

### Conclusions

The results of our study indicate that sleep disorders, particularly SMA, are common in children with NMD. Furthermore, our examination of parental stress levels revealed a high level of stress among these parents, which can affect the quality of care for their children. Therefore, interventions should be implemented to improve the sleep of these children. Additionally, due to the high level of stress experienced by parents, it is necessary to implement measures for screening, identification, and referral for counseling. These families should be regularly evaluated and supported, and interventions should be tailored based on the intensity of their stress.

## Abbreviations

CSHQ: Children’s Sleep Habits Questionnaire
NMD: neuromuscular disorder
SDB: sleep-disordered breathing
SMA: spinal muscular atrophy
SRI: Stress Response Inventory
